# CIDE Proteins in Human Health and Disease

**DOI:** 10.3390/cells8030238

**Published:** 2019-03-13

**Authors:** Mark Slayton, Abhishek Gupta, Bijinu Balakrishnan, Vishwajeet Puri

**Affiliations:** Department of Biomedical Sciences and Diabetes Institute, Ohio University Heritage College of Osteopathic Medicine, Athens, OH 45701, USA; slayton@ohio.edu (M.S.); guptaa2@ohio.edu (A.G.); balakrib@ohio.edu (B.B.)

**Keywords:** CIDEA, CIDEB, FSP27, fat metabolism, lipid droplets, adipose, liver

## Abstract

Cell death-Inducing DNA Fragmentation Factor Alpha (DFFA)-like Effector (CIDE) proteins have emerged as lipid droplet-associated proteins that regulate fat metabolism. There are three members in the CIDE protein family—CIDEA, CIDEB, and CIDEC (also known as fat-specific protein 27 (FSP27)). CIDEA and FSP27 are primarily expressed in adipose tissue, while CIDEB is expressed in the liver. Originally, based upon their homology with DNA fragmentation factors, these proteins were identified as apoptotic proteins. However, recent studies have changed the perception of these proteins, redefining them as regulators of lipid droplet dynamics and fat metabolism, which contribute to a healthy metabolic phenotype in humans. Despite various studies in humans and gene-targeting studies in mice, the physiological roles of CIDE proteins remains elusive. This review will summarize the known physiological role and metabolic pathways regulated by the CIDE proteins in human health and disease.

## 1. Introduction

Maintenance of an energy exchange balance is important to all forms of life. CIDE proteins (Cell death-inducing DNA fragmentation factor alpha-like Effector) are an important group of proteins that were originally identified as pro-apoptotic proteins [1,2]. Several years after their discovery and classification as pro-apoptotic proteins, the CIDE proteins were unexpectedly identified as lipid droplet-associated proteins [3,4,5]. This association is a direct physical interaction with the surface of the lipid droplet, as well as with other lipid droplet proteins, such as perilipin. There are three known CIDE proteins—CIDEA, CIDEB, and CIDEC (herein referred to as FSP27)—which share the homologous CIDE-N domain and the CIDE-C domain, which varies between them. The tissue expression pattern of each CIDE protein differs but is correlative with the fact that they associate with lipid droplets—CIDEA is found primarily in brown adipose (human and mouse) or white adipose tissue (humans only), CIDEB is highly expressed in the liver, and FSP27 is predominately found in white adipose tissue [1,3,5,6,7]. Disruption of the normal expression of CIDE proteins in mice and humans results in varying metabolic phenotypes between the two species. Several recent studies involving the CIDE proteins have emerged with data from in vitro experiments, as well as in mouse models and in human patients, but the results have been conflicting [6,8,9,10,11,12,13]. It is now clear that the CIDE proteins play an important role in the regulation of lipid homeostasis.

## 2. Role of CIDE Proteins in Apoptosis

Originally, the CIDE proteins were discovered as apoptotic proteins through a homology search targeting the CIDE-N domain of DNA fragmentation factor 45 (DFF45). The apoptotic pathway is highly regulated and the prevention of its normal activation is linked to several disease states, including cancer [14]. Near the end of the apoptotic pathway, DNA is condensed and then fragmented by the nuclease DNA fragmentation factor 40 (DFF40). However, DFF40 is maintained as a complex with DFF45, which inhibits DFF40 until it is cleaved by upstream-activated caspases. The interaction between DFF40 and DFF45 occurs at the N-terminal of both proteins, termed the CIDE-N domain. This domain was utilized in a homology search and other CIDE-N domain-containing proteins were identified [1]. FSP27 (Fat-specific protein, 27 kDa) was technically the first of the CIDE proteins to be discovered by characterization of cDNA clones derived from highly-expressed genes during adipocyte differentiation [15]. However, it was not until the identification of CIDEA and CIDEB, via homology to the CIDE-N domain, that the alternative names “CIDEC” or “CIDE-3” were used when referring to FSP27 [1,16]. Each of the CIDE proteins share homology with the N-terminal of DFF45 to varying degrees (CIDEA, 39%; CIDEB, 29%; FSP27, 38%) and interact with DFF45 at this conserved domain [1,2]. DFF45 inhibits the apoptotic action of the CIDE proteins through this domain in a manner similar to its interaction with DFF40.

The apoptotic role of CIDE proteins has been primarily demonstrated through ectopic overexpression in mammalian cells or through induced mutations to different amino acid residues of the individual CIDE proteins [1,16,17]. Initially, the apoptotic functions of the CIDE proteins were thought to be independent of caspase activation [1]; however, further studies revealed that this function is dependent on caspase-3, caspase-9, and the release of cytochrome c [18,19]. In mice, FSP27 forms a homodimer through interactions at the CIDE-N and CIDE-C domains; additionally, mouse FSP27 forms a heterodimer with CIDEA but this interaction occurs at the CIDE-C domain only [2,19]. Recently, CIDE-domain containing proteins (Drep2, Drep4, DFF40, FSP27) were discovered to form head-to-tail helical oligomers, a confirmation that is important for their function [20]. In line with the above observations, both the rate of apoptosis and the level of CIDEA expression were markedly increased in skeletal muscle following an induced burn injury in mice [21], the CIDE-C domain of CIDEB interacts with the hepatitis C non-structural protein 2 (NS2) in the liver to prevent apoptosis [18], and the region of the human genome containing FSP27 (chromosome 3p25) was mutated or absent in several forms of human cancers [16].

For the CIDE proteins to induce apoptosis and fragment DNA, they must localize to the mitochondria [16,17]. This notion was challenged by Puri et al. when they identified that FSP27 and CIDEA are highly expressed in adipocytes [5,22] where they are localized to lipid droplets. They found that these proteins were not localized to mitochondria, rather they were associated with lipid droplets and that the endogenous proteins have a crucial role in lipid metabolism [5,22,23]. Thus, these proteins were recognized as lipid droplet associated proteins.

A set of elegant studies were performed by Cynthia Smas’ group, in which they discovered a dual role for FSP27 in adipocyte metabolism and cell death. FSP27 is highly upregulated during adipogenesis and is dependent on the differentiation of preadipocytes; the FSP27 transcript is downregulated by the presence of tumor necrosis factor-α (TNF-α), but it is upregulated by the presence of insulin [24]. Additionally, this group confirmed that FSP27 did not localize to the mitochondria of COS cells. They overexpressed mouse FSP27 ectopically in 293T and 3T3-L1 cells, which resulted in a cellular morphology indicative of apoptosis; however, there was a lack of DNA fragmentation in 3T3-L1 cells, suggesting that the presence of endogenous lipid droplet machinery inhibits the apoptotic action of FSP27 [24]. Furthermore, in HeLa cells treated with oleic acid, ectopic FSP27 promoted the formation of lipid droplets but did not induce apoptosis [19].

Overall, the role of CIDE proteins in terms of apoptosis has not yet been fully elucidated, but several required factors have been identified. Taken together, these studies indicate that the CIDE proteins are involved in the DNA fragmentation step of the apoptotic cascade; however, there remains much to be discovered in this area of research. Since their discovery as lipid droplet-associated proteins, much of the work regarding the CIDE proteins has been shifted towards investigating their roles in lipid metabolism.

## 3. Discovery of CIDE Proteins as Lipid Droplet-Associated Proteins

FSP27 was discovered 25 years ago as an adipocyte-specific gene that is upregulated during adipogenesis [15,25] and is highly expressed in both white and brown adipose tissues [26]. It was identified to be a differentiation-regulated protein activated by peroxisome proliferator-activated receptor-γ (PPARγ) [27]. However, the question of FSP27’s function remained unsolved until 15 years later, when Puri et al. identified that FSP27 associates with lipid droplets and modulates lipid droplet function for optimal storage of triglycerides by adipocytes [5]. Subsequently, these results were complemented by studies from various other groups [28,29]. A follow-up study showed that the CIDE proteins share significant homology [7] and that CIDEA is also associated with lipid droplets in humans. Thus, the CIDE-domain containing proteins are lipid droplet proteins that define a novel, highly-regulated pathway of triglyceride deposition in human white adipose tissue. Failure of the triglyceride storage pathway results in ectopic lipid accumulation, insulin resistance, and its associated comorbidities in humans. A later study by Ye et al. demonstrated that CIDEB, a liver specific protein, localizes to the endoplasmic reticulum (ER) and lipid droplets, where it promotes the formation of very low-density lipoprotein (VLDL) particles [30]. Overall, these studies suggested a role for CIDE family proteins in lipid metabolism.

## 4. CIDE Proteins and Lipid Metabolism

It appears that depending on the cell type and level of expression, the CIDE proteins localize to additional intracellular organelles. In the case of CIDEA, localization to the Golgi apparatus occurs, while CIDEB is targeted preferentially to the smooth ER. VLDL is transported from the ER in vesicles which contain CIDEB; the biogenesis of these vesicles is also regulated by CIDEB [31,32,33]. FSP27 is the only CIDE protein that has yet to be observed in the ER, although its presence has been speculated in the ER [34].

Each of the three CIDE proteins localize to the surface of lipid droplets in their respective tissue types (CIDEA, white and brown adipose tissue; CIDEB, liver; FSP27, white adipose tissue) [35,36,37,38,39]. CIDEA directs lipid droplet transfer and fusion in brown and white adipose tissue of mice, as well as in other cell types when expressed ectopically [35,38]. CIDEB was found to promote the storage of lipids in the liver of mice while on a regular diet, but CIDEA and FSP27 increased the size of the droplets under high-fat conditions [36]. Interestingly, in addition to the homology with the CIDE-N domain of DNA fragmentation factor 45, both CIDEA and FSP27 contain four homology domains with perilipin and both localize to the surface of lipid droplets in 3T3-L1 adipocytes [5,7]. Two independent studies demonstrated that FSP27 promotes the enlargement or fusion of lipid droplets via clustering and lipid transfer [37,39]. Perilipin1 interacts with FSP27 during lipid droplet fusion at the CIDE-N domain, but not with CIDEA or CIDEB, suggesting that this interaction is specific within white adipose tissue [34,40]. Interestingly, the CIDE-N domain of FSP27 is dispensable for lipid droplet enlargement, but the CIDE-C domain is required [41]. In concurrence with these results, silencing of FSP27 results in a higher number of small lipid droplets, while overexpression increases their size [37,39]. FSP27 expression in the liver is primarily through an alternative transcript, FSP27β, which is regulated by cyclic-AMP-responsive-elemental-binding protein H (CREBH), a transcription factor primarily expressed in the liver [42]. Compared to the main FSP27 isoform, FSP27α, which is transcriptionally regulated by PPARγ and enriched in white adipose tissue, FSP27β contains 10 additional N-terminal amino acids and is involved in the development of hepatic steatosis. Interestingly, although FSP27α is positively associated with unilocular lipid droplets in white adipocytes, FSP27β was recently found in brown adipocytes and decreases lipid droplet size through a regulatory interaction with CIDEA [43]. These data suggest that the tissue localization of each CIDE protein acts as an additional factor in their function, along with intracellular localization.

As regulators of lipid droplet dynamics, the CIDE proteins are also involved in the rate of intracellular lipolysis. Through the use of RNA interference, Nordström et al. disrupted CIDEA in human preadipocytes and observed an increase in lipolysis and secretion of (TNF-α) [4]. However, the depletion of CIDEA in adipocytes did not change the rate of lipolysis, demonstrating a difference between the use of preadipocytes and adipocytes [9], while the depletion of FSP27 had a marked increase. It was further demonstrated that FSP27 protects against TNF-α-mediated lipolysis, inhibits adipose triglyceride lipase (ATGL) through a protein-protein interaction, and suppresses the ATGL promoter in conjunction with early growth response transcription factor (EGR1) [44,45,46]. In hepatocytes, CIDEB is transcriptionally regulated by PPARγ-coactivator-1-alpha (PGC-1α) and positively correlated with lipid droplet size, which in turn causes the downregulation of adipose differentiation-related protein (ADRP) [47]. This process can be induced by treatment with human serum and results in enhanced VLDL secretion. These studies demonstrate that the CIDE proteins are important regulators of lipid metabolism by controlling both the size of lipid droplets and the rate of their lipolysis.

## 5. Role of CIDE Proteins in Mouse Physiology

Several knockout studies targeting the CIDE proteins and their metabolic effects have been performed in mice. Mice deficient in Cidea (Cidea^−/−^) have a lean phenotype, higher metabolic rate and body temperature, enhanced lipolysis in brown adipose tissue, and are resistant to diet-induced obesity and diabetes [6]. Mechanistically, this study suggested a direct interaction of Cidea with uncoupling protein 1 (UCP1) to inhibit its thermogenic potential. However, Cidea was later identified not to be localized in the mitochondria. Another potential mechanism was identified in that Cidea interacts with AMP-activated protein kinase (AMPK) -β subunit and stimulates ubiquitination-mediated degradation of AMPK [48]. In Cidea-knockout mice, the loss of Cidea-enhanced AMPK stability in brown adipose tissue results in higher fatty acid oxidation and energy expenditure, which contribute to the development of a lean phenotype. Interestingly, the transgenic expression of human Cidea in mouse adipose tissue resulted in a higher body weight than the wild-type littermates, but with improved insulin sensitivity and the maintenance of a healthy obese phenotype [11]. These studies warrant further work to determine whether human and mouse Cidea have opposite roles, or if there is a secondary effect of Cidea knockout that causes increased fatty acid oxidation. A putative explanation could be that enhanced lipolysis in the absence of Cidea would increase availability of FFAs as substrates of fatty acid oxidation. 

The tissue-specific expression of Cidea plays a critical role in the healthy or unhealthy phenotype observed in mice. Cidea expression is undetectable in the liver of normal mice but its level is significantly upregulated by treatment with PPAR α and γ ligands [49]. Promoter analysis of the Cidea gene revealed that it contains three putative proliferator response elements that are responsive to both PPAR isotypes. Sterol-regulatory-element-binding protein 1c (SREBP1c) mediates the fatty acid synthesis in liver upon insulin stimulation [50]. In mice, liver Cidea expression was trivially reduced upon fasting and restored on refeeding. Furthermore, a sterol regulatory element (SRE) was found in the Cidea promoter region; ChIP analysis confirmed the binding of SREBP1c to the SRE. It was demonstrated that the insulin-mediated effect on Cidea expression was regulated by SERBP1c in the mouse liver. Cidea expression is increased in the hepatocytes of alcohol-induced fatty liver mice and the acetaldehyde concentration in the serum of these mice was significantly increased [51]. Acetaldehyde directly induced the expression of Cidea and was mediated via SERBP1c.

Free fatty acids are well known to induce apoptosis in pancreatic β-cells [52]. It was found that Cidea expression was significantly elevated during palmitic acid induced apoptosis in mouse pancreatic β-cells [53]. In addition, silencing of Cidea alleviated palmitic acid-induced apoptosis. The suppression of forkhead box protein O1 (FOXO1) prohibited the upregulation of Cidea in palmitic acid induced apoptosis, suggesting that Cidea is a downstream target of FOXO1.

In addition to adipocytes, liver, and pancreas, Cidea was shown to be highly expressed in sebaceous glands, where the deficiency of Cidea leads to dry hair and hair loss in aged mice [54]. Cidea was found in lactating mammary glands, where it acts as a co-activator of CCAAT/enhancer-binding protein (C/EBP-β) and regulates lipid secretion in milk; the lack of Cidea in the milk of nursing dams can lead to the premature death of their pups [55].

A limited number of studies targeting Cideb in mice have been performed. Cideb is highly expressed in the liver. Apart from the liver, Cideb is expressed in the kidney, stomach, small intestine, colon, and in pancreatic beta cells [10,33]. Cideb localizes to the surface of lipid droplets and the ER, where it promotes the lipidation and maturation of VLDL by interacting with apolipoprotein B (apoB) [30]. In the small intestine, Cideb plays a crucial role in lipidation of chylomicrons [33]. Nascent VLDL particles are transported from the hepatic ER through an ER-specialized vesicle called the VLDL transport vesicle (VTV); knockdown of Cideb in mice has shown to reduce VTV biogenesis significantly [30]. Consequently, mice deficient in Cideb have reduced levels of blood triglycerides (TGs) and non-esterified fatty acids (NEFA) [3]. When fed with a high-fat diet, these mice maintained a lean phenotype and were resistant to hepatic steatosis. They also displayed an increased rate of body metabolism and insulin sensitivity. The resistance to high-fat diet-induced obesity in Cideb-deficient mice was the result of downregulation of fatty acid synthesis through SREBP1c, a crucial factor for TG synthesis. These studies suggested that Cideb might have a negative impact on metabolic health in mice; however, a recent study by Chao et al. manifested that Cideb deficient mice were more vulnerable to dextran sulfate sodium (DSS)-induced ulcerative colitis [56]. DSS induces more oxidative stress in colonocytes and was more deteriorative when the Cideb-deficient mice were fed with a high-fat diet.

Similar to Cidea and Cideb, mouse models of Cidec (herein referred to as Fsp27) have given mixed responses in health and disease. Whole body Fsp27-deficient mice have reduced levels of TGs and smaller lipid droplets in their white adipocytes and were protected from diet-induced obesity [28], resulting in higher glucose uptake rates and improved insulin sensitivity. Furthermore, Fsp27-deficiency led to improved metabolic rates by upregulating genes involved in mitochondrial oxidative metabolism and browning of white adipose tissue [13]. Similarly, silencing of Fsp27 using antisense oligonucleotides in high-fat fed mice and ob/ob mice resulted in decreased visceral adiposity, improved insulin sensitivity, and whole body glycemic control [57]. Srijana et al. found that in mouse adipocytes, TNFα-mediated lipolysis is associated with downregulation of Fsp27 [45]. Conversely, lipolysis mediated by isoproterenol was accompanied by upregulation of Fsp27 levels via a delay of the ubiquitin-dependent proteosomal degradation pathway. Complementing these results, Tanaka et al. confirmed that adipose-specific Fsp27 knockout mice were resistant to high fat diet-induced weight gain [58]. These mice had small white adipose mass with multilocular lipid droplets in their adipocytes. The impairment of lipid storage function in adipocytes resulted in ectopic fat accumulation and led to hepatosteatosis and systemic insulin resistance. The above studies suggest that Fsp27 affects whole body insulin sensitivity in a tissue-specific manner.

PPARγ agonism is used clinically to reduce insulin resistance and improve hyperglycemia associated with type 2 diabetes [59], however, this treatment markedly elevates TG levels in the liver associated with a number of murine models of diabetes or obesity [13,60]. Matsusue et al. showed that Fsp27 in the liver of ob/ob mice is a direct target gene of PPARγ to elevate hepatic triglyceride levels [61]. Though there might be intrahepatic insulin resistance in these mice, the whole-body insulin sensitivity was presumably increased due to the therapeutic effect of the PPARγ agonist. Adipocyte-specific disruption of Fsp27 causes hepatosteatosis and insulin resistance in high-fat diet-fed mice, indicating that PPARγ agonist-induced expression in adipose tissue might predominate the effect of fatty liver in terms of whole-body metabolism. A later study showed that Fsp27β, but not Fsp27α, is induced in the liver of ob/ob and other mouse models of fatty liver disease in a CREBH-dependent manner [42]. The above contradictory studies warrant further analysis of Fsp27’s role in the liver and its tissue-specific role in whole body insulin sensitivity.

Nonetheless, the role of liver-specific Fsp27 expression has consistently been shown to cause fat accumulation in the liver. Hepatocyte-specific disruption of Fsp27 in mice provided protection from the development of alcoholic steatohepatitis [62]. Similar results were obtained when mice were treated with adenovirus-Fsp27shRNA. Therefore, upregulation of Fsp27 in the liver contributes to alcohol-induced liver damage. Fsp27, presumably Fsp27α, has been shown to play a crucial role in the formation of unilocular large lipid droplets in white adipocytes. Fsp27β isoform, besides the liver, is expressed in the brown adipose tissue but in a CREBH-independent manner [43]. Fsp27β and Cidea co-operatively form small multilocular lipid droplets, which is an advantageous morphology for the transport of FFA to mitochondria for oxidation in brown adipocytes. Since CREBH is not expressed in brown adipose tissue, the mechanisms responsible for regulating the expression of Fsp27β in brown adipose tissue remain unclear.

Studies on fasting mice showed that Fsp27 expression in the liver is initially increased, however in the later stages of fasting, the expression was decreased [63]. This early response of Fsp27 was contributed to by the PKA (protein kinase A)-CREB-CRTC2 (CREB-regulated transcription coactivator 2) signaling pathway. This study is in line with the role of Fsp27 in the regulation of lipolysis [44,46], since during the late stages of fasting, lipolysis is increased to keep up with the supply of fatty acids and energy production.

An exciting role of Fsp27 in osteoarthritis has been identified recently [64]. High-fat diet-induced osteoarthritis models revealed that FFA released from lipid droplets exerted the lipotoxic effect observed in articular chondrocytes. Articular chondrocytes were rescued from FFA-induced lipotoxicity by FFA sequestration, which was mediated by protein kinase casein kinase 2 (PKCK2), a six-transmembrane protein of prostate 2 (STAMP2) and Fsp27.

Cidea/Fsp27 double-deficient mice exhibited a drastic reduction in lipid storage capabilities, reflected by smaller lipid droplets in white adipose tissue compared to Cidea or Fsp27 single-deficient mice [38,65]. In these studies, the Fsp27-deficient mice lost the ability to store lipids in their adipose tissue, which resulted in ectopic storage of fat in the liver and insulin resistance. Conversely, Cidea/Fsp27 double-deficient mice did not have ectopic storage of lipids in the liver and exhibited improved insulin sensitivity. Fsp27-deficient mice were mated with leptin (ob/ob) knockout mice to obtain double deficient ob/ob/Fsp27^−/−^ mice [65]. These mice showed significantly less body weight compared to the littermate controls due to a lower accumulation of both visceral and subcutaneous fat, as well as a substantial reduction in adipose inflammation and increased adiponectin level. Plasma glycerol levels in ob/ob/Fsp27^−/−^ mice were drastically elevated compared to littermate controls due to increased lipolysis; these mice also developed fatty liver and insulin resistance. Overall, these studies demonstrated that a lack of Fsp27 expression in mice significantly reduces the lipid storage capabilities of adipose tissue and results in ectopic fat deposition that leads to hepatic steatosis and insulin resistance.

**Figure 1:**
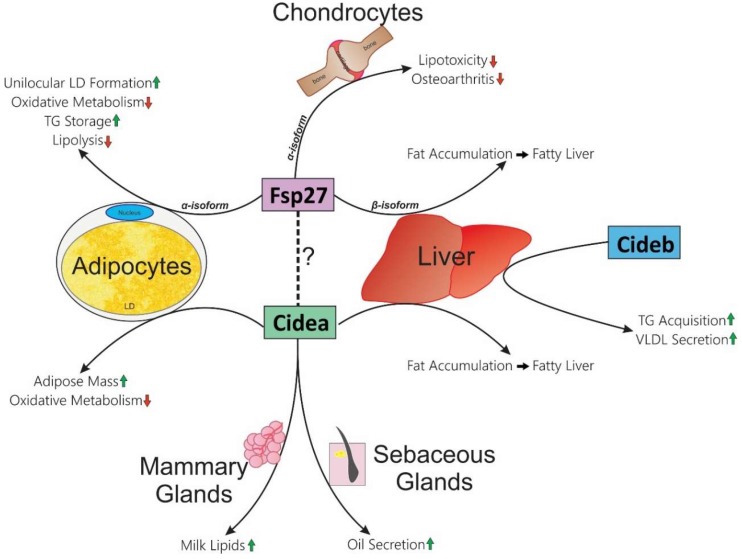
Tissue-specific functions of CIDE proteins in mice. Each CIDE protein is indicated with colored rectangles. The known functions within respective tissues are listed at the end of arrows which pass by them (Cidea: adipocytes, liver, mammary glands, and sebaceous glands; Cideb: liver; Fsp27: adipocytes, chondrocytes, and liver). Green up-arrows indicate an increase; red down-arrows indicate a decrease. Dotted line indicates that Fsp27 and Cidea could form a heteromeric complex, which could be essential for one or more of their functions.

In conclusion, the metabolic effects observed in various mouse models of CIDE proteins have not been consistent with their positive versus negative role in health and disease (Figure 1). It could be that complete knockout or exogenous expression of these proteins produces secondary effects in mice that contributes to the outcomes observed. Furthermore, the tissue-specific expression of CIDE proteins appears to have differential effects on whole-body metabolism.

## 6. Role of CIDE Proteins in Human Metabolic Health

Gene association studies (also known as polymorphism studies) focus on variations amongst specific genes and alleles and their association with disease. According to the National Center for Biotechnology Information (NCBI), around 6658 single nucleotide polymorphisms (SNPs) have been found in the human CIDEA genes, although not all of them are associated with the disease phenotype. Worldwide clinical studies report around 66 SNPs in CIDEA that are associated with human diseases (NCBI ClinVar). Of these 66 SNPs, 24 are deletions, whereas 42 are duplications.

The human CIDEA gene is located on chromosome 18 (18p11) and spans around 23.22 kilobases (kb) in length, with 4 introns and 5 exons. Linkage studies performed to assess the association of body mass index reported the association of this locus with the development of obesity [66]. Another independent study performed by Parker et al. determined that the chromosome 18p11 region is positively associated with type 2 diabetes and obesity [67].

**Figure 2:**
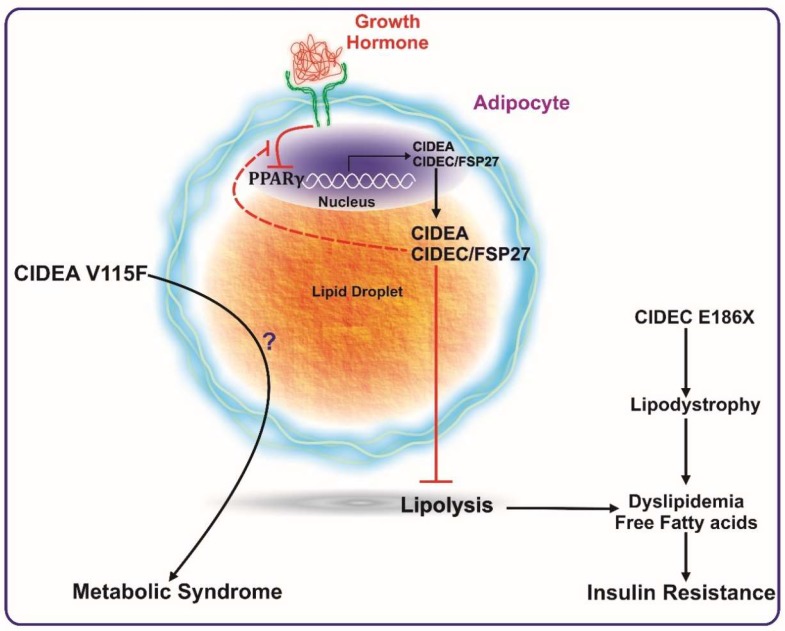
CIDEA and CIDEC/FSP27 are transcriptionally activated by PPARγ expression. CIDEA and FSP27 bind to lipid droplets and inhibit lipolysis. Reduction in PPARγ activation (such as by growth hormone) reduces the expression levels of these proteins, which ultimately causes enhanced lipolysis, leading to a higher concentration of systemic free fatty acids. The increase in serum free fatty acids culminates in insulin resistance. A mutation in FSP27 (CIDEC E186X) causes lipodystrophy and insulin-resistant type 2 diabetes. The V115F polymorphism in CIDEA is reported to be positively associated with metabolic syndrome. Excess growth hormone levels (such as in acromegaly patients) inactivate PPARγ causing reduced expression of FSP27. FSP27 protects against GH-mediated destabilization of PPARγ to maintain insulin sensitivity.

A study by Dahlman et al. revealed the association of a CIDEA polymorphism (V115F) with the occurrence of obesity (see Figure 2) [68]. This study was performed in two different Swedish cohorts comprising men and women categorized into non-obese and obese populations. Characterization of the V115F polymorphism revealed six different polymorphic sites in obese individuals, as compared to the non-obese counterparts. However, only one SNP (C19878G→T) was found in the coding region and was responsible for creating the V115F mutation. The study concluded that this polymorphism was positively and significantly associated with obesity and reduced basal metabolic rate. An additional study comprised of 272 male Japanese individuals identified the same polymorphism (V115F) [69]. This study assessed the association of this amino acid substitution) with the prevalence of metabolic syndrome between two groups—VV vs. VF + FF (valine, V; phenylanaline, F). They concluded that VF + FF individuals have higher abdominal obesity, high fasting plasma glucose levels, and an increased risk of metabolic syndrome. Similarly, an independent study in a Chinese cohort clarified the positive association of the CIDEA V115F polymorphism with the risk of metabolic syndrome [8]. 

Another study conducted by Wu et al. studied the association of five polymorphisms in the CIDEA gene with obesity in the Chinese population [70]. The polymorphisms included were rs1154588/V115F, rs4796955, rs8092502, rs12962340, and rs7230480. Individuals with the rs1154588/V115F, rs4796955, rs8092502, or rs7230480 polymorphism had around 1.4-fold increased risk of developing obesity. Further, the study concluded that individuals with both rs1154588/V115F and rs4796955 polymorphisms were more prone to developing diabetes. In addition to the above SNPs, the association of rs2479 and rs1053239 SNPs in CIDEA with rapid progression of high blood pressure have been reported [70]. The individuals with rs2479 SNP are more prone to having elevated fasting blood glucose levels along with high triglyceride levels. Overall, the above polymorphism studies unequivocally showed the positive association of CIDEA with healthy metabolic phenotypes in humans.

Functionally, CIDEA was identified as a lipid droplet-associated protein, and its expression was in consonance with insulin sensitivity in obese humans [7]. In this study, Puri et al. confirmed the PPARγ-mediated regulation of CIDEA expression in white adipose tissue samples from obese individuals undergoing gastric-bypass surgery (see Figure 2). CIDEA expression in white adipose tissue of obese human subjects was determined and found to correlate positively with whole-body insulin sensitivity independent of gender or BMI. Diet-induced weight loss showed a positive correlation of CIDEA and metabolic health [71,72]. Interestingly, changes in adipose tissue CIDEA mRNA levels paralleled variations in insulin sensitivity independently of the change in body mass index [72]. CIDEA was up-regulated in the adipose tissue of individuals with successful long-term insulin resistance relapse and not in adipose tissue of unsuccessful individuals, suggesting a beneficial role of adipose tissue CIDEA in long term glucose homeostasis, independently of weight variation.

Similar to CIDEA, a specific SNP in FSP27 has also been shown to be associated with the causation of metabolic syndrome. The SNP (G→T) occurs at nucleotide position 556 within exon 6 of FSP27 and inserts a premature stop codon (TAA), resulting in a non-sense mutation (E186X) that disrupts the CIDE-C domain. This mutation has been reported to be associated with partial lipodystrophy and insulin resistance in a female patient (see Figure 2) [12].

Homeostatic Model Assessment for Insulin Resistance (HOMA-IR; an index for assessment of insulin sensitivity) is positively correlated with FSP27 expression in BMI-matched obese individuals [7]. The level of FSP27 in adipose tissue reduces with the development of obesity and is negatively correlated with TNF-α [73]. Complimentarily, FSP27 expression was found to be decreased with increasing BMI, fasting glucose, fasting insulin, and HOMA-IR [74]. The latter study elegantly showed that bariatric surgery-induced weight loss caused an increase in FSP27 expression in subcutaneous adipose tissue in parallel to adipogenic and mitochondrial genes. Interestingly, PLIN1 levels were also increased. The latter observation was in line with a positive correlation of adipose PLIN1 expression with insulin sensitivity in obese humans [7]. Various other studies have suggested the role of PLIN1 in metabolic health in humans [75], indicating a concerted effort of these lipid droplet-associated proteins in maintaining insulin sensitivity.

FSP27 regulates lipid droplet dynamics and lipolysis in adipocytes through regulation of the catalytic capacity [44], as well as through transcriptional regulation of adipose triglyceride lipase (ATGL), the rate-limiting enzyme in lipolysis [46]. These studies demonstrated a mutual relationship between FSP27 and ATGL in regulating lipolysis, triglyceride accumulation, and insulin signaling in human adipocytes. As a proof of concept, the former study showed that FSP27 protects human adipocytes from FFA-mediated insulin resistance [44], thus highlighting the mechanistic role of FSP27 in maintaining insulin sensitivity, potentially by maintaining an optimal balance of energy storage and breakdown.

Recently, the role of FSP27 in human growth hormone (GH)-induced diabetes was elucidated [76]. GH-induced lipolysis in human subjects is tightly associated with an acute reduction in CIDEC expression in subcutaneous adipose tissue. Furthermore, the study described that GH-induced lipolysis is mediated via Mitogen-activated protein kinase kinase/Extracellular signal-regulated kinase (MEK/ERK) activation, which suppresses PPARγ transcriptional activity (see Figure 2) with a feedback loop, by which FSP27 protects PPARγ from being phosphorylated at Ser^273^. PPARγ Ser^273^ phosphorylation has been linked with the development of insulin resistance [77,78,79]. In human adipocytes exposed to GH, exogenous FSP27 stabilized PPARγ in the nucleus by inhibiting its phosphorylation at Ser^273^ [76]. Another complimentary study in mice and mouse cell lines showed that GH modulation of FSP27 expression is mediated through activation of both MEK/ERK and STAT5 dependent intracellular signaling, whereby, GH-induced MEK/ERK pathway has a predominating role in PPARγ inactivation [80]. Overall, these studies deciphered part of the molecular mechanism by which FSP27 regulates insulin sensitivity.

Taken together, the various mouse models have shown contradictory results on the role of CIDE proteins in whole-body metabolic phenotype. On the contrary, all of the human studies published to date unequivocally show a negative association of CIDEA and FSP27 with the pathogenesis and pathophysiology of metabolic disease in humans (Figure 2), giving prominence to their role in improving the metabolic health of humans.

## 7. Concluding Remarks

The studies reported here indicate that the CIDE proteins are important regulators of fat metabolism. The functionality of each CIDE protein appears to depend highly on intracellular localization and tissue expression. It is currently unclear as to why there is a discrepancy between the metabolic phenotypes from studies targeting CIDE proteins in mice versus humans. However, the studies agree that the CIDE proteins are involved in lipid storage. Furthermore, the metabolic consequences of tissue-specific CIDE protein knockout appears to be different than that of whole-body knockouts. The human studies published to-date unequivocally show a negative association of CIDEA and FSP27 with the pathogenesis and pathophysiology of metabolic disease in humans. Further work is required to elucidate the mechanism by which the expression of each CIDE protein contributes to the regulation of insulin sensitivity or resistance and to examine their use as a potential therapeutic.
